# Linear Discriminant Analysis Achieves High Classification Accuracy for the BOLD fMRI Response to Naturalistic Movie Stimuli

**DOI:** 10.3389/fnhum.2016.00128

**Published:** 2016-03-31

**Authors:** Hendrik Mandelkow, Jacco A. de Zwart, Jeff H. Duyn

**Affiliations:** Advanced MRI Section, Laboratory of Functional and Molecular Imaging, National Institute of Neurological Disorders and Stroke, National Institutes of HealthBethesda, MD, USA

**Keywords:** BOLD fMRI, multivariate pattern analysis (MVPA), classification, movies, naturalistic stimuli, linear discriminant analysis (LDA), Gaussian Naïve Bayes (GNB), nearest-neighbor

## Abstract

Naturalistic stimuli like movies evoke complex perceptual processes, which are of great interest in the study of human cognition by functional MRI (fMRI). However, conventional fMRI analysis based on *statistical parametric mapping* (SPM) and the *general linear model* (GLM) is hampered by a lack of accurate parametric models of the BOLD response to complex stimuli. In this situation, statistical machine-learning methods, a.k.a. multivariate pattern analysis (MVPA), have received growing attention for their ability to generate stimulus response models in a data-driven fashion. However, machine-learning methods typically require large amounts of training data as well as computational resources. In the past, this has largely limited their application to fMRI experiments involving small sets of stimulus categories and small regions of interest in the brain. By contrast, the present study compares several classification algorithms known as Nearest Neighbor (NN), Gaussian Naïve Bayes (GNB), and (regularized) Linear Discriminant Analysis (LDA) in terms of their classification accuracy in discriminating the global fMRI response patterns evoked by a large number of naturalistic visual stimuli presented as a movie. Results show that LDA regularized by principal component analysis (PCA) achieved high classification accuracies, above 90% on average for single fMRI volumes acquired 2 s apart during a 300 s movie (chance level 0.7% = 2 s/300 s). The largest source of classification errors were autocorrelations in the BOLD signal compounded by the similarity of consecutive stimuli. All classifiers performed best when given input features from a large region of interest comprising around 25% of the voxels that responded significantly to the visual stimulus. Consistent with this, the most informative principal components represented widespread distributions of co-activated brain regions that were similar between subjects and may represent functional networks. In light of these results, the combination of naturalistic movie stimuli and classification analysis in fMRI experiments may prove to be a sensitive tool for the assessment of changes in natural cognitive processes under experimental manipulation.

## Introduction

Over the past two decades *functional magnetic resonance imaging* (fMRI) based on the *blood oxygenation level dependent* (BOLD) contrast mechanism has become the neuroimaging tool of choice for brain research in humans. In spite of this success, the statistical analysis and interpretation of BOLD fMRI data with the goal of understanding brain function remains a daunting task that continuously motivates the development of new and improved analytical methods. Conventional *hypothesis-driven* fMRI analysis of task-evoked activity involves an explicit model of the BOLD fMRI response to the stimulus. Relevant stimulus features must be identified *a priori* and transformed by an equally predetermined *hemodynamic response function* (HRF) to form the design matrix of a *general linear model* (GLM). By contrast, data-driven *machine-learning* (ML) methods, often referred to as *multivariate pattern analysis* (MVPA) in the context of fMRI, are attractive, because they do not require any generative model of the BOLD signal. ML algorithms for *clustering, decoding*, and especially for *classification* facilitate the quantification of stimulus-related information without making strong assumptions about the nature of the BOLD fMRI signal (Kriegeskorte et al., 2006; Norman et al., 2006; Pereira et al., 2009). Even without a generative model of the fMRI signal such information can serve to detect relevant differences between perceptual, cognitive or pathological brain states (e.g., in a brain-machine interface; Naci et al., 2012; Sorger et al., 2012; Yuen et al., 2012) or between experimental conditions (e.g., to evaluate experimental sensitivity; Chen et al., 2015).

fMRI data analysis by means of non-parametric, model-free ML methods is particularly interesting in conjunction with *naturalistic* stimuli like photographs or movies, because these are intended to evoke the complex perceptual processes of everyday life, for which adequate fMRI signal models are lacking despite unique research in the field (Nishimoto et al., 2011; Horikawa et al., 2013). In this situation, exploratory data analysis techniques are expected to generate new testable model hypotheses. Indeed, machine-learning algorithms may offer the best chance of finding widely applicable models of the BOLD fMRI signal reflecting higher cognitive functions.

Since watching TV is a common cognitive task in modern society, motion pictures may be regarded as naturalistic stimuli, even though cinematographic fiction is not always a faithful representation of real life. Motion pictures are not only naturalistic but also convenient stimuli, because they are easy to obtain and to deliver in a controlled fashion. Important for the success of classification analysis is the fact that movies can elicit a robust and widespread fMRI response that is consistent across experimental runs and between subjects. It has been argued that naturalistic stimuli often elicit an fMRI response that is more *reproducible*, although not necessarily stronger than purely artificial visual stimuli like gratings used in traditional vision research (Hasson et al., 2010). This increase in fMRI signal reproducibility could be mediated largely by the global effects of changes in attention, as captivating movies that attract attention were found to result in higher inter-trial correlations than less *exciting* footage (e.g., from a surveillance camera). This is not implausible, since attention is known to have a strong modulatory effect in cognitive experiments (e.g., Çukur et al., 2013).

In the recent literature, fMRI data from movie-viewing experiments have mostly been analyzed by conventional (univariate) GLM analysis, which quantifies correlations of the fMRI signal with scalar regressors derived from visual features such as optical flow (motion), luminance or the presence of faces in the movie (e.g., Bartels et al., 2008; Huth et al., 2012; Russ and Leopold, 2015). Alternatively, fMRI signal correlations between repeated experimental runs have been used as a (model-free) measure of fMRI signal reproducibility in specific brain regions (Hasson et al., 2004; Jääskeläinen et al., 2008). Two published studies have applied classification methods in conjunction with naturalistic movie stimuli, but neither discuss the performance of different algorithms (Haxby et al., 2011; Chen et al., 2015). At the same time a limited number of studies have compared classification algorithms [notably the *support vector machine* (SVM), *linear discriminant analysis* (LDA), and *Gaussian Naïve Bayes* (GNB)] for the analysis of fMRI data, but all of those focused on binary classification, a small number of stimulus categories such as houses and faces, and input from small predefined brain regions of just a few hundred voxels (Ku et al., 2008; Misaki et al., 2010; Churchill et al., 2014; Yourganov et al., 2014). According to these studies the performance of ML methods depends not only on the choice of classification algorithm but also crucially on the pre-processing of the data, which must be tailored specifically to the type of fMRI experiment. It is therefore uncertain that classification methods designed and optimized for binary classification (like the SVM) are directly applicable to the more general classification problem with a large number of classes and a high-dimensional feature space (e.g., >10^4^ voxels in the brain).

The purpose of this study is to compare several ML methods suitable for the classification of BOLD fMRI data evoked by large sets of naturalistic stimuli, which are presented as a 5-minute movie and repeated a number of times. Such data pose a challenge for classification algorithms, because they are characterized by a high-dimensional feature space (≫1000 voxels) and a large number of more or less distinctive classes (>100 stimulus time points) that far exceeds the number of experimental repetitions (< 10 stimulus presentations). In this work, several suitable classification methods were therefore systematically evaluated to find a maximum in classification accuracy, which is a proxy for the information content of the fMRI signal. Given the conceptual proximity between classification methods and clustering algorithms this line of research may eventually bridge the gap between data-driven and hypothesis-driven fMRI analysis by revealing the relevant stimulus features underlying the fMRI signal in natural conditions.

## Methods

### fMRI experiments

#### fMRI stimuli and paradigm

Four subjects (age 24 ± 1.2 years, 2 male) each gave written informed consent in accordance with institutional guidelines at the NIH (IRB-approved protocol 00-N-0082) to participate in a series of fMRI experiments. During each fMRI experiment the iconic opening scene from the popular action movie “The Matrix” was presented for exactly 5 min (video without audio). This kind of naturalistic stimulus was intended to elicit a broad range of perceptual and cognitive processes thus precipitating a varied fMRI response, suitable for analysis by machine-learning (ML) classification algorithms. Experiments were repeated over 4–6 separate scan sessions spread over several months until each subject had completed eight viewings of the same video stimuli, 1–2 per session depending on time constraints.

#### fMRI data acquisition

MRI experiments were performed on a high-field 7 T MRI scanner (Siemens, Erlangen, Germany) equipped with 70 mT/m @ 200 T/m/s gradients and a combined 2-channel transmit 32-channel receive head coil (Nova Medical, Wilmington, USA). ${\text{T}}_{2}^{*}$-weighted single-shot EPI images in transversal orientation were acquired with a TR of 2 s and an isotropic spatial resolution of 2 mm. Other sequence parameters typical for BOLD fMRI at 7 T included: TR/TE = 2 s/25 ms, FA = 63°, BW = 2004 Hz/pixel, matrix = 96 × 96, GRAPPA = 3. As an anatomical reference for the co-alignment of fMRI time series a whole-brain volume of ${\text{T}}_{2}^{*}$-weighted images at 1 mm isotropic resolution was acquired by a GRE sequence (TR/TE = 3 s/24 ms; FA = 80°; BW = 230 Hz/pixel; matrix = 256 × 192).

A 3 cm gap between the outer transmit and inner receive coil accommodated a mirror and a small MR-compatible CCD camera (MRC Systems GmbH, Heidelberg, Germany) to allow both the projection of visual stimuli and video recording of the subject's eye movements using eye-tracking software by Arrington Research, Inc. (USA). A separate computer running the Psychophysics Toolbox (Brainard, 1997; http://psychtoolbox.org) in Matlab served to play the digital video stimuli in synchrony with MRI acquisition triggers and the eye tracker. In addition, each subject's cardiac and respiratory signals were recorded by pulse-oxymetry and a pneumatic chest belt.

### fMRI data analysis

Previous studies of fMRI during movie stimulation have mostly focused on either inter-trial correlations as a measure of reproducibility (e.g., Hasson et al., 2008, 2010; Jääskeläinen et al., 2008) or (manually) extracted regressors like motion energy or the presence of faces in the movie to perform a classical GLM analysis (e.g., Bartels and Zeki, 2004; Huth et al., 2012; Russ and Leopold, 2015). By contrast, the current study applied classification methods to movie-fMRI data in order to quantify the *information content* of the fMRI signal. This approach was inspired by the inter-subject correlations proposed by Haxby et al. (2011), but is more closely related to a recent study by Chen et al. (2015), which also focuses exclusively on intra-subject classification thereby avoiding the fundamental problem of inter-subject alignment. The following analysis treated each fMRI volume acquisition related to one particular time point in the movie (*t* = n^*^TR) as a separate stimulus category for classification, even though some stimuli might be more similar than others in terms of the evoked BOLD fMRI response. Unlike other studies, this work was not aimed at determining what visual features drive the fMRI response and how the brain might categorize stimuli. Instead classification accuracy, i.e., the percentage of correctly identified test stimuli, served us purely as a measure of information extracted from the fMRI signal and therefore classifier performance.

#### fMRI data pre-processing

Pre-processing and analysis of the fMRI data were performed using custom code^1^ written in Matlab supplemented by tools from AFNI (http://afni.nimh.nih.gov; Cox, 1996) and FSL (http://fsl.fmrib.ox.ac.uk; Smith et al., 2004). fMRI data from all experimental runs underwent slice-timing and motion correction, they were aligned to each subject's individual T_2_-weighted anatomical scan and resampled to an isotropic resolution of 1.2 mm. To preserve resolution, all spatial operations were combined into a single linear transformation with a 7-point sinc interpolation for re-gridding. Motion parameters as well as linear and quadratic trends were regressed out before converting each voxel's time series to a *z*-score by subtracting the mean and normalizing the variance over time.

#### Feature selection by univariate ANOVA

**Figure 1:**
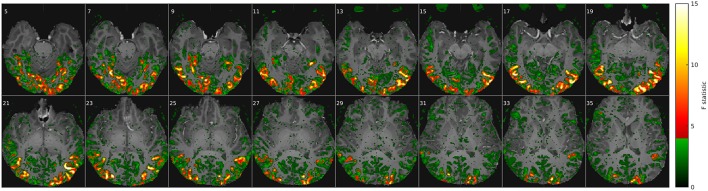
**Movie stimuli elicited a widely distributed fMRI response throughout the brain**. Example of a voxel-wise ANOVA F-statistic map (threshold *p* < 1%, uncorrected) superimposed on the T_1_-weighted anatomical MRI of one representative subject (16 axial slices in radiological convention). Stimulus-correlated fMRI activity is widespread in the *occipital* and *ventral temporal cortices*, consistent with their established involvement in basic vision and object recognition. Red/yellow colors mark the top 2^14^ voxel features that maximized classification rates in **Figure 2**.

Even though ML algorithms are in principle designed to extract relevant information from noisy data, the performance of such classifiers on high-dimensional data generally depends on *feature selection* as an initial step to exclude input data of high noise and low signal content. To avoid making assumptions about the number and location of voxels (i.e., features) that are most informative for classification, we repeated all classification analyses for feature sets ranging from 16 to 2^16^ voxels, selecting those that were most significantly modulated by the stimulus. The relative *significance* (or *fMRI activation strength*) in each voxel location was estimated by a univariate analysis of variance (ANOVA), treating each of the 150 fMRI volume acquisitions during the movie as an independent group (or *factor*) and each experimental run as a repeated measurement (Figure 1). The resulting functional region of interest (fROI) was variable in size and computed only from the training data in each round of cross-validation. This was done just to be orthodox, since the location of the fROI and the resulting classification accuracy varied only marginally with or without cross-validated feature selection. In any case, the results below show that optimal classification rates were contingent on a large fROI size between 4000 and 20,000 voxels and were not sensitive to small changes.

#### Classification methods

The multi-class classifiers compared here are in essence all based on the efficient *nearest-neighbor* (NN) or *nearest-mean* (NM) classification scheme and evaluated by leave-one-experiment-out cross-validation. In other words, any sample pattern (from the test data) is assigned the same class as its nearest neighbor from a set of class templates (from the training data) as determined by a metric of pairwise distances. Because systematic differences between fMRI scans constitute a large part of the noise confounding classification, the cross-validation scheme strictly avoided sharing data from any one experiment between training and validation data. Without this leave-one-*experiment*-out cross-validation scheme, scan specific variations e.g., in shimming, coil sensitivities, subject performance or even breathing patterns are likely to outweigh inter-stimulus differences especially for similar stimuli close in time. In other words, different samples (classes) from the same fMRI scan were often more similar than samples from the same class in different scans.

Table 1 details the distinguishing features of all classifiers compared in this study. Essentially they differ in the distance metric employed, which is either the correlation distance (one minus the Pearson correlation coefficient) or a (normalized) Euclidean distance with various constraints imposed on the estimate of the normalizing noise covariance matrix. As indicated in Table 1, some of these classifiers are commonly referred to as GNB and LDA. The statistical model underlying both of these common classifiers is a mixture of multivariate Gaussian distributions, but with different degrees of freedom.

**Table 1:** **Summary of classifiers compared**.

**Legend Figure 2**	**PCA components**	**Nearest-Neighbor (NN) Nearest-Mean (NM)**	**Distance metric**	**Distance metric *D(y, x, Σ) =***
NNC	–	NN	Correlation distance	$1-\frac{{\vec{y}}^{T}\vec{x}}{\left||\vec{y}||||\vec{x}|\right|}$
NNE	–	NN	Euclidean distance	$\left||\vec{y}-\vec{x}|\right|$
NMC	–	NM	Correlation distance	
NME	–	NM	Euclidean distance	$\sqrt{\underset{n}{\sum }{\left(y_{n}-\mu_{n}\right)}^{2}}$
GNB	–	NM	norm. Euclidean distance	$\sqrt{\underset{n}{\sum }\frac{{\left(y_{n}-\mu_{n}\right)}^{2}}{\Sigma_{nn}}}$
NMC 8, 16, 32,…	8, 16, 32,…	NM	Correlation distance	
NME 8, 16, 32,…	8, 16, 32,…	NM	Euclidean distance	
GNB 8, 16, 32,…	8, 16, 32,…	NM	norm. Euclidean distance	
LDA 8, 16, 32,…	8, 16, 32,…	NM	Mahalanobis distance	$\sqrt{{\left(\vec{y}-\vec{\mu }\right)}^{T}\Sigma^{-1}\left(\vec{y}-\vec{\mu }\right)}$

y, x: sample vectors of test and training data; y_n_, x_n_: components of y, x, μ: class mean (centroid) vector from training data; Σ_nn_: diagonal elements of the covariance matrix Σ of the (pooled) training-data residuals (x−μ).

The simplest classifier tested here can be described as pairwise nearest-neighbor classification with each (single) experimental run in turn serving as a set of class templates (training data), against which every fMRI volume from any other experimental run (test data) was compared either by the Euclidean or the correlation distance (Table 1, NNE + NNC). More effective were the various forms of LDA based on multivariate Gaussian distribution models that represent each stimulus class by its mean vector (*centroid*) and a covariance matrix estimated from the residuals of its members (after subtracting the class mean). Variants of *discriminant analysis* are typically qualified as *linear* or *quadratic* as well as GNB, all of which refer to constraints imposed on the estimated model covariance matrix: Linear and quadratic discriminant analysis respectively refer to a common (*pooled*) or a class-specific (*stratified*) estimate of the within-class (noise) covariance matrix. Similarly, the GNB classifier assumes a diagonal noise covariance matrix (i.e., uncorrelated voxels or features)—a simplifying assumption that is not necessarily accurate, but convenient and often surprisingly effective in situations where a small number of samples in a high-dimensional input space renders the covariance matrix rank deficient and not invertible.

**Figure 2:**
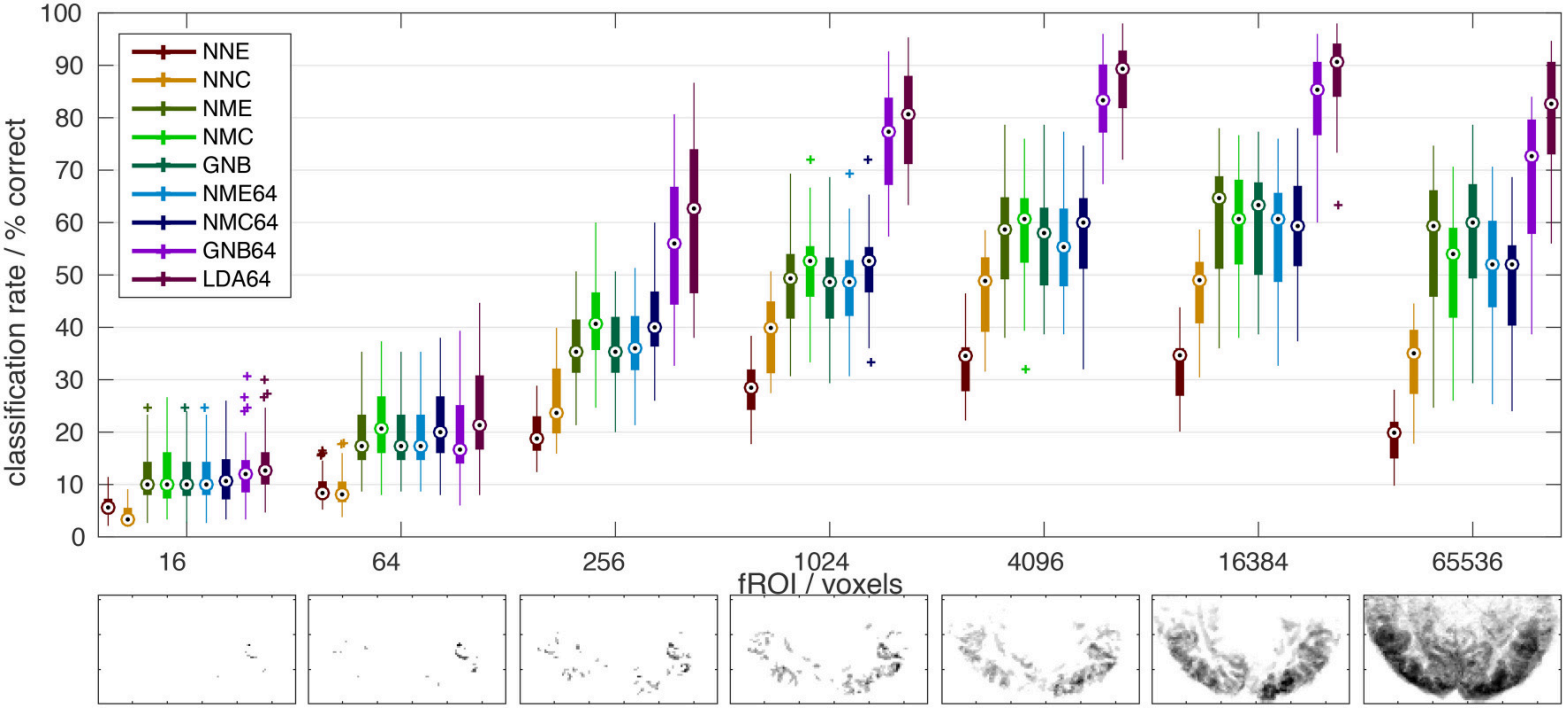
**Comparison of classifier performance**. **(Top)**: Bar-plot summary of classification rates averaged over subjects and cross-validation runs (4 × 8): Each bar marks the median (dot), the 25–75% inter-quartile range (bar), and the full data range (line), as well as any outliers exceeding 1.5x the inter-quartile range (+). Classification rates depended strongly on the number of input voxels (x-axis) and the classification algorithm (color labels, see Table 1): NNC, NNE: *Correlation* and *Euclidean distance* metrics used in pairwise *nearest-neighbor* classification; NMC, NME, GNB: *Correlation, Euclidean, and normalized Euclidean distance* (= GNB) metrics combined with *nearest-mean* classification; NMC64, NME64, GNB64, LDA64: Same as preceding classifiers after dimensionality reduction to 64 PCs plus LDA based on the *Mahalanobis distance* metric. **Bottom**: The growing extent of the functional ROI in one subject is illustrated by axial maximum intensity projections (sum over slices) of the voxels included.

**Figure 3:**
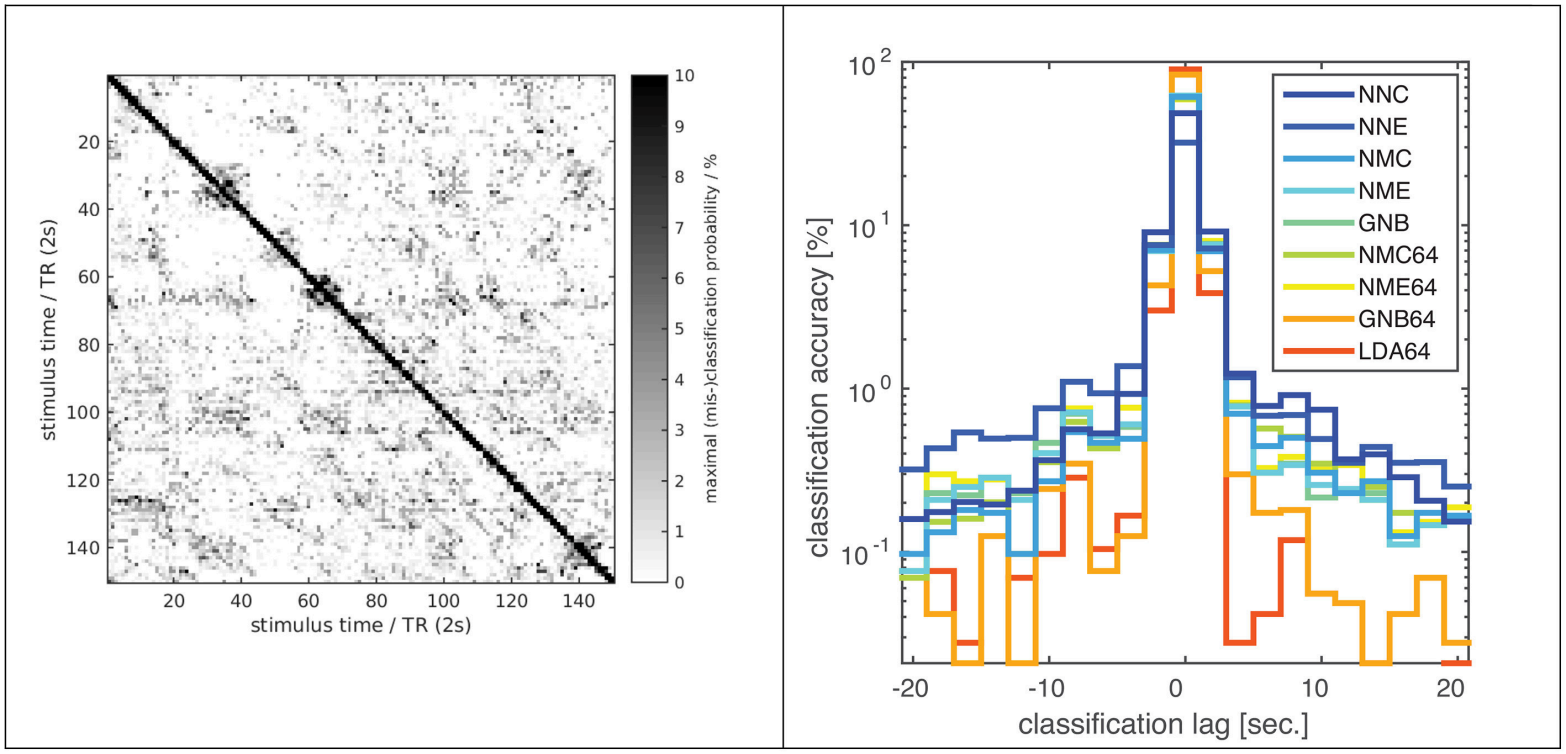
**Classification error distribution in time**. A confusion matrix (**left**) of (mis-)classification rates between training and validation stimuli (x/y axes) averaged over all subjects, experiments and classifiers (based on the optimal input fROI of 2^14^ voxels) and the corresponding (mis-)classification lag histogram (**right**) of the same data both illustrate that most mis-classifications fall within ±1 TR (2s) of the correct target simulus (i.e., the diagonal = lag zero; Classifier labels NNC, NNE, etc. refer to Table 1). The known hemodynamic lag of the BOLD signal offers an obvious explanation, but the visible block-diagonal structure (**left**) implicates the compounding effect of perceptual similarity between stimuli. Some blocks of high similarity apparently correspond to scenes of fast motion or prominent faces in the movie stimulus.

**Figure 4:**
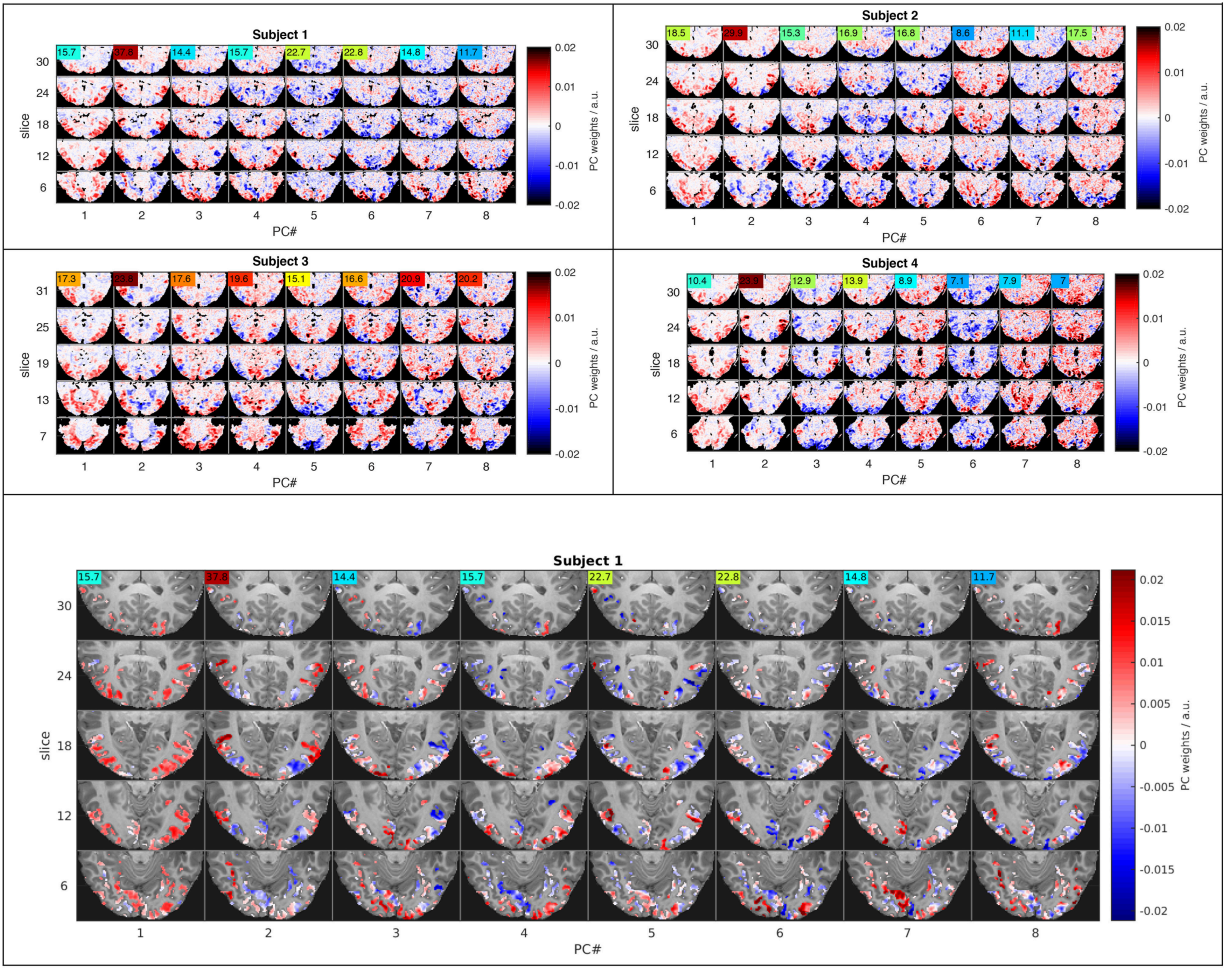
**Functional connectivity patterns revealed by the eight leading principal components**. Five representative slices (rows) representing projections of the eight highest-variance PCs (columns) in four subjects (small top panels) illustrate similarities across subjects and large-scale interactions between major subdivisions of the visual system. The ANOVA F-statistic (colored numbers) for each PC time course indicates, which components strongly influence LDA classification in PC space. The large bottom panel shows the feature space used for PCA in the context of the T_1_-weighted anatomy of the first subject.

Another common solution to this *curse-of-dimensionality* problem is dimensionality reduction by *principal component analysis* (PCA), which was employed here as a form of regularization to facilitate LDA based on the Mahalanobis distance that requires a full-rank covariance matrix. All the nearest-mean LDA-type classifiers were tested by leave-one-experiment-out cross-validation, meaning that PCA, class centroids and covariance matrices were computed from training data that included all but one experimental run, while the left-out experiment served as validation data for testing classification results. The comparisons in Figures 2, 3 include only classifiers using 64 principal components. This number of PCs yielded the highest classification accuracies in an extended comparison of classification results computed for 8, 16, 32, 64, and 128 PCs (Figure 6 in Supplementary Material). Note that PCA was performed on the training data *after* averaging over experiments. An alternative computation of PCA on training data concatenated in time resulted in a much larger number of eigenvectors, some apparently specific to individual experiments, others obviously non-neuronal (e.g., emphasizing the ventricles). To compute sample distances and classification results, both the training and validation data were projected into the *spatial* PCA eigenbasis of the *training* data. Note that the resulting signal time series are not normalized in variance, unlike the temporal PCA eigenbasis. Figure 4 shows five representative slices of the first 8 PCs computed from an (optimal) fROI of 2^15^ voxels in each of the four subjects. For display purposes only, the *temporal* PCs estimated from the fROI were projected back to the *whole* fMRI volume, such that any correlations outside the fROI would also be observed.

## Results

### Eye tracking

The eye-tracking data confirm a large degree of consistency in the way subjects watch the movie stimuli, notwithstanding a considerable amount of variance due to technical limitations. Eye blinks, head motion and subject non-compliance, i.e., fluctuations in vigilance, are major sources of variability between experimental runs and subjects. Obvious manifestations of subject drowsiness are extended intervals of partial or complete eye closure and corresponding periods of fMRI signal that is inconsistent with other experimental runs. As a result, subjects, who were less able to maintain vigilance over a number of experimental runs, showed lower average classification accuracy. Nevertheless, after removing obvious artifacts (global drifts, discontinuities, out-of-bounds signal) the eye traces recorded at 30 Hz exhibit a high degree of correlation across experimental runs and between subjects. (Pearson correlation between experimental runs typically >0.5, data not shown.) In fact, an overlay of gaze positions from multiple experiments onto the dynamic movie stimulus makes it obvious that there are rarely more than one or two foci of attention at any given moment during the movie.

### ANOVA F-statistic maps

The ANOVA F-statistic maps from a typical subject (Figure 1) clearly delineate consistently activated regions in the occipital and inferior temporal cortices, including areas that are commonly associated with the *ventral visual pathway* of high-level object recognition. Functional ROIs of adjustable size were defined on these maps by selecting between 16 and 2^16^ (65,536) of the most *activated* voxels, i.e., those showing the highest F-values. For reasons of computational efficiency, this input ROI for classification analysis was confined to the occipital half of the fMRI volume, where virtually all the stimulus-correlated fMRI signal was concentrated. By contrast, frontal regions close to the paranasal sinuses were strongly affected by susceptibility artifacts as well as a stimulus-correlated motion artifact in the eyes, which corroborates the consistent eye-tracking results and would certainly confound stimulus classification.

### Classification accuracy

The main results of this study are summarized in Figure 2, which compares all classifiers (color labels, refer to Table 1) in terms of their mean classification rate (y-axis) and as a function of the number of input features i.e., voxels in the fROI (x-axis). All classifiers performed well above the theoretical chance level (1/150 = 0.7%), but the classification accuracy depended strongly on the classification algorithm and the extent of the fROI. Irrespective of their vastly different peak classification rates between 30 and 90%, all classifiers showed a similar functional dependence on the fROI size with a broad maximum between 4000 and 20,000 voxels, roughly 5–25% of all voxels showing significant activity in these experiments [p(F) < 1% uncorrected]. This is the expected consequence of first adding voxels of high functional contrast (F-statistic) and later voxels that contribute less signal than noise.

A similar dome-shaped dependence was observed when testing classification rates as a function of dimensionality reduction using 8–150 principal components (Figure 6 in Supplementary Material), although a peak was only reached for the Mahalanobis and normalized Euclidean distance metrics and contingent on the fROI size. The reason might be the limit on the maximal number of PCs, which cannot exceed the number of stimulus time points in these experiments. With the inclusion of more PCs, Euclidean and correlation distances computed in PC space should approximate the equivalent voxel-space analyses, since PCA is an orthonormal transform. In any case, the number of PCs is an influential tuning parameter, which was set at 64 (out of 150 maximum) for the highest classification rates in the final comparison.

At their maxima (~16,000 voxels), the LDA and GNB classifiers based on *averaging, PCA*, and *covariance weighting* achieved the highest classification rates (~90% and ~85% respectively). By comparison, the nearest-mean classifiers (NMC, NME, GNB, Table 1) based on averaged class templates performed considerably worse, close to 60%, irrespective of the distance metric. Notably, this was still higher than pairwise nearest-neighbor classification of single-trial fMRI data without any averaging (~30–50% for NNE + NNC, respectively), which yielded particularly poor results with the Euclidean distance metric (NNE).

### PCA maps

**Figure 5:**
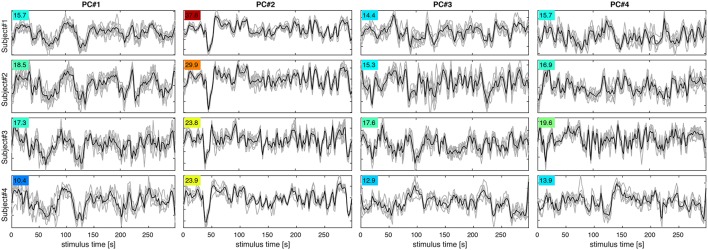
**PC signal time courses for PC#1–4 (columns) in subjects 1–4 (rows)**. Each panel shows PC signals for eight experimental runs (gray) and their mean (black). The F-statistic (colored numbers) quantifying signal over noise variance for each set of curves indicates, which PCs are most reproducible and likely to contribute to classification success.

Dimensionality reduction by PCA in combination with GNB or LDA strongly increased classification rates in our experiment. It was therefore of interest to investigate the spatial basis functions that account for most of the signal variance as well as their within-class noise covariance. Both of these determine each component's weighting as part of the normalized Euclidean and the Mahalanobis distance metrics. The basis functions computed by PCA can be viewed as spatial brain maps (Figure 4) that indicate, which brain regions are positively or negatively correlated with the associated signal time course (Figure 5).

Figure 4 shows maps of the eight leading (i.e., highest-variance) PCA components (spatial PCs), a few representative slices from each subject. Following convention, the components are sorted in the order of descending variance contribution (PC#1–8). However, the PCs of highest variance need not be the ones contributing most reliably to classification success. This is indicated by an ANOVA F-statistic quantifying the ratio of *between-class* over *within-class* variance for each temporal PC (colored numbers in Figures 4, 5). This suggests an alternative strategy for selecting the most relevant PC features. Finally, one may appreciate the degree of anatomical similarity between the leading spatial PCs from different subjects as well as the corresponding PC time courses.

While this study is not concerned with the relationship between specific stimuli and the spatial distribution of the fMRI response, we will briefly comment on spatial characteristics of the PCs observed. Since PCA is equally sensitive to spatially correlated signals of neuronal and non-neuronal origin (e.g., subject motion or scanner drift), it is reassuring to note that all of the leading PCs reflect the known functional anatomy of the visual cortex and other functionally related brain regions. Notably, there is a first PC common to all subjects that broadly covers lateral occipital and ventral temporal cortices. The second common component highlights the motion sensitive area V5/hMT and has the highest F-statistic in all subjects. Many of the following PCs (#3, 4,…) seem to contrast different sub-regions of the visual cortex V1-4, which one would expect to be represented by a number of PCs. In general, the leading PCs exhibit a clustered structure of low spatial frequency and bilateral symmetry that resembles functional networks reported in studies of task-based and resting-state functional connectivity (e.g., Smith et al., 2009).

## Discussion

The present study demonstrates the use of cinematographic videos presented repeatedly as visual stimuli (without sound) for the purpose of reliably eliciting a widespread, diverse, and reproducible BOLD fMRI signal response in human subjects. Consistent with previous studies, the naturalistic movie stimuli evoked reproducible activity in the occipital and ventral temporal cortex (Golland et al., 2007; Hasson et al., 2008; Jääskeläinen et al., 2008). These brain areas have been described as *object selective* and as part of the *ventral visual stream*, which is also known as the *What pathway* that processes the semantic identity of visual percepts (review Grill-Spector and Weiner, 2014). In contrast to other studies, the present analysis is not concerned with the relationship between stimulus content and the activation of specific brain regions (Huth et al., 2012). Instead, we tested and optimized the ability of various ML classification methods to detect and discriminate stimulus-specific fMRI signals. The identification of reliable and efficient algorithms is an important prerequisite to establishing the use of classification methods as an alternative, data-driven and information-based strategy for analysing BOLD fMRI data from naturalistic movie viewing experiments. In light of the present results, such methods have the potential to detect perceptual and cognitive changes in individual subjects. It is important, however, to understand what aspects of the data acquisition and processing strategy determine experimental sensitivity.

Previous fMRI studies have applied and compared classification methods mostly based on experiments with few distinct stimulus categories presented in a block-design or event-related paradigm (Ku et al., 2008; Yourganov et al., 2014). For such data the linear SVM was generally found to yield superior classification performance. However, the popular SVM is ill suited to handling the large number of classes in the data at hand. For this reason, our study compared several multi-class classification methods related to LDA, in essence all nearest-neighbor (NN) classifiers using a variety of distance metrics. The classifiers compared all performed well above chance given a sufficient number of input voxels. In fact, all classifiers showed *qualitatively* equivalent results, performing just above chance level for fewer than 16 voxels and reaching peak performance broadly between 4000 and 20,000 voxels (about 5–25% of all activated voxels) before declining again with higher voxel counts. The rise, peak and fall of classification rates as a concave function of functional ROI size is more than plausible under the assumption that the voxel features added first are highly informative, while those added later are apt to confound classification with a higher noise contribution and less signal reproducibility as evidenced by lower values of the F-statistic. Since all classifiers consistently benefited from a large fROI one may also conclude that stimulus-related information in the brain is spatially distributed and its coarse global structure most reliably interpreted.

Notwithstanding these *qualitatively* similar results, Figure 2 reveals substantial *quantitative* differences between classifiers. Since average classification rates mark a lower bound on the information content of the BOLD fMRI signal, higher classification rates amount to higher sensitivity to detecting stimulus-driven information, and better performance when utilizing such information e.g., in a diagnostic test. In theory, classifiers that model the signal and noise statistics of the data more accurately would show higher classification rates. Therefore, differences in the performance of various classifiers may offer insight into the analyzed signal and its noise structure.

Firstly, we note that the correlation distance metric performed substantially better than the Euclidean distance for pairwise classification with individual scans (rather than class averages) as templates (Figure 2, NNE + NNC). Secondly, nearest-mean (NM) classifiers based on averaged class templates achieved higher classification accuracy (>60%) than pairwise nearest-neighbor classification (< 50%). Interestingly, the distance metric had little influence in this regime judging by the equivalent results for the Euclidean, normalized Euclidean and correlation distance (Figure 2, NME, GNB, NMC).

Detailed inspection of pairwise cross-classification rates between individual experiments (NNE, NNC) confirmed that classification accuracy varied and tended to be higher when training and validation data were acquired during the same scan session (Figure 7 in Supplementary Material). For this reason the cross-validation scheme chosen here avoided sharing any data from the same experiment or scan session between training and validation data. Such findings are not uncommon (Misaki et al., 2010): In spite of successful co-alignment, signal mixing through interpolation and partial-volume effects retains some dependence on the original scan geometry, shimming, slice timing, etc. not to mention a subject's daily constitution. Such experimental variations were mitigated by averaging 6–7 volumes from different scans. One might also suspect implicit spatial smoothing to mediate such effects, but explicit Gaussian smoothing of the data to 3 mm FWHM did not improve classification rates (data not shown). In any case, data acquisition and cross-validation schemes are important caveats to consider when evaluating and comparing fMRI classification rates across scans or studies.

Finally, in addition to the use of mean class templates, another leap in classification accuracy was achieved by the combination of PCA and LDA, which respectively amounts to utilizing the covariance matrices of both the (mean) signal and the residuals (after subtraction of the class means). Note that PCA alone did not confer any increase in classification accuracy, although it did offer a 100-fold reduction in dimensionality (~64/16,000) without substantially compromising results (compare Figure 2 NME + NME64). However, subsequent normalization by the estimated within-class noise covariance (that factors into the normalized Euclidean and Mahalanobis distance metrics for GNB and LDA) increased classification accuracy by 20–30%. In effect this amounts to weighting PCs by scaling down those harboring most of the *intra-class* noise variance. This variance normalization made the number of PCs another critical optimization parameter, as results deteriorated again with an increasing number of low-variance PCs (Figure 6 in Supplementary Material). Incidentally, the number of 32–64 PCs that maximized classification rates in our experiments seems to confirm similar experiments by Haxby et al. (2011) and is comparable to the number of functional connectivity networks that are typically distinguished by PCA and ICA in the field of resting-state fMRI (Abou Elseoud et al., 2011; Ray et al., 2013). One may wonder, if this reflects some intrinsic dimensionality of the fMRI signal, although that may well depend on SNR and spatial resolution.

The highest classification accuracy of 90% on average was achieved by the PCA-regularized LDA classifier based on the Mahalanobis distance. This classifier required regularization or dimensionality reduction by PCA, since the necessary covariance matrix of the residuals is inevitably rank-deficient and not invertible in the original high-dimensional data space. Note that the GNB classifier based on a diagonal approximation to the residual covariance matrix in the same eigenbasis yielded just marginally lower classification accuracies around 85%. This would indicate that off-diagonal cross-terms played a subordinate role in the residual covariance matrix. In other words, signal and noise components were largely separated by PCA. Notably, the same GNB classifier afforded no increase in classification rate when applied without PCA in the original data space, where voxel correlations are known to be high. As one would expect, the first 16–32 PCs of highest variance accounted for most of the classification accuracy. Still, at least 64 PCs of diminishing variance contributed to increasing classification accuracies. Of course, components carrying more noise than signal are selectively suppressed by LDA and GNB classifiers. The F-statistic for each PC (Figure 4, color labels) indicates, which PCs are most relevant under the covariance-weighting scheme. Note that the first PCs of highest variance are not the most relevant according to the F-statistic.

The observed smooth tuning functions for the fROI size and the number of extracted PCs might be somewhat misleading in the sense that they portray a continuous, gradual and progressive accumulation of information, which might in fact be less distributed than it seems. The sequential feature selection scheme based on the F-statistic implemented here may be effective but not necessarily optimal in an information theoretic sense. In other words, algorithms for selecting and combining a subset of fMRI signals to achieve optimal stimulus discrimination with a limited number features represents another promising area of investigation. However, the exploration of (computationally expensive) feature-selection algorithms is beyond the scope of this paper that focuses on a comparison of classification methods.

The component vectors of PCA should be viewed as spatial maps of correlated (fMRI) signal sources (Figure 4). One might expect these to reflect networks of functionally connected and co-activated brain regions, however, stimulus-correlated artifacts and residual noise components are also likely to be captured in some components. Note that the subject-wise PCA analysis performed here makes no attempt at finding components that are common *between* subjects; such *hyperalignment* methods are not directly pertinent to this study (Chen et al., 2014). Nevertheless, many of the leading principal components exhibit a temporal and anatomical structure that is similar between subjects and strongly reminiscent of functional connectivity patterns described in other contexts (e.g., Smith et al., 2009).

The first PC in all subjects reflects a global unison activation of cortical regions accounting for the largest fraction of variance but not the highest F-statistic. This PC may well reflect a system-wide modulatory effect like arousal. The second PC exhibits the highest F-statistic indicating the most reproducible stimulus response in all subjects. This and the apparent involvement of motion-sensitive areas V5/hMT suggests that PC#2 reflects optical flow and motion, which are known to account for a large proportion of fMRI signal variance throughout the visual system (Bartels et al., 2008; Nishimoto et al., 2011; Russ and Leopold, 2015). Other leading PCs accentuate various parts of the occipital visual cortex likely representing differential activity in areas V1–V4, which would be expected to encode much of the visual stimulus variations.

The leading 16–32 PCs, which account for most of the fMRI signal variance and classification accuracy, extend over large areas of the brain. This could mean that widely distributed regions across the brain are under the direct influence of a common signal source, e.g., global visual features like luminance and motion or stimulated arousal. However, it has been argued (Hasson et al., 2010) that the commonly observed functional connectivity patterns with large-scale regional variations is more consistent with another popular hypothesis, which postulates a number of distinct networks of interconnected brain regions as the major processing units of the brain. Our results, showing the best classification accuracies for extensive functional ROIs, primarily confirm that information about the identity of diverse visual stimuli is widely distributed across the occipital and temporal cortices.

Few studies have systematically compared classifiers in fMRI experiments with static visual stimuli (Ku et al., 2008; Misaki et al., 2010; Pereira and Botvinick, 2011; Yourganov et al., 2014) and literally only a couple of studies have applied *within-* or *between-subject* classification to fMRI data from dynamic movie stimulation (Haxby et al., 2011; Chen et al., 2015). The present study combines both approaches, and its results are broadly consistent with the literature, judging by the reported classification accuracies and the relative ranking of different classifiers. However, a direct comparison between studies is problematic and agreement not necessarily expected, given substantial differences in experimental procedures and data processing. As illustrated above, nominal classification rates are highly sensitive to a number of factors, including the extent of input patterns in voxel space and any form of averaging over space or time, not to mention any perceptual similarity between the presented stimuli themselves. In this regard one must, for instance, distinguish studies that perform classification on *statistical parametric maps* (SPMs) generated by a preceding GLM analysis, which is sometimes termed an *encoding model* and constitutes a form of averaging that constrains the ML model (Misaki et al., 2010; Naselaris et al., 2015). For the kind of non-randomized dynamic data generated in movie-viewing experiments, classification rates can also be boosted by extending input patterns over several TR in time, which will help disambiguate very large data sets (Haxby et al., 2011). In the present experiments most classification errors are in fact misclassifications of fMRI volumes adjacent in time, i.e., separated by only one TR of 2 s (Figure 3). This might be plausibly explained by the well-known hemodynamic response function, which has a FWHM of 5–6 s and causes strong autocorrelations on that time scale in the fMRI signal. However, the overall high classification rates and a noticeable block diagonal structure in the confusion matrix (Figure 3) suggest that perceptual stimulus similarity plays a major role: The blocks of more similar (i.e., confusable) stimuli actually correspond to movie scenes of high motion content or prominent faces. Also, the overall high classification rates indicate that BOLD auto-correlations do not preclude distinctive BOLD signal patterns, presumably because of the highly consistent sequence and timing of movie stimuli. In the same vein, a recent study using 30-s video stimuli in conjunction with fast fMRI acquisitions by means of the *simultaneous multi-slice technique* found that the number of discriminable fMRI signal patterns increased with decreasing TR even as short as 600 ms before saturation was reached (Chen et al., 2015).

Finally, it should be mentioned that there is technically no guarantee that classification in experiments like these is driven by neuronal phenomena. Especially with experiments that do not allow for a counterbalanced randomized stimulation paradigm, a caveat one must consider with ML techniques is the possibility that correlated artifacts like systematic scanner drifts or stimulus-correlated motion could underlie classification success. In this study we consider this unlikely, because the PCs are reminiscent of functional networks, and because within-subject cross-classification between two separate movie stimuli yielded classification rates at chance level (data not shown).

The present study confirms the suitability of several classification methods for fMRI experiments with movie stimuli and it demonstrates the separate influences of the distance metric and the feature space (fROI) on absolute classification rates. In analogy to previous work (Haxby et al., 2011; Chen et al., 2015), we plan to use this experimental paradigm to evaluate the sensitivity of fMRI acquisition and pre-processing techniques. Conversely, we also envisage the use of short fMRI experiments with movie stimuli as calibration scans for comparing the BOLD fMRI signal across scan sessions and subjects. Eventually, the examination of distance and confusion matrices as well as the noise covariance in relation to stimuli and other metadata may reveal new information about the underlying physiological and cognitive processes. For example the experimental paradigm should lend itself to the study of cognitive habituation effects, although the analysis will have to be modified to hone in on systematic inter-scan variations rather than similarities. This is work in progress. The present analysis primarily supports the conclusion that some aspects of the human BOLD fMRI response remain (surprisingly) stable over several viewings of the same movie stimulus.

## Conclusions

The results presented above demonstrate how fMRI experiments with repeated naturalistic movie stimuli can suitably be analyzed using multivariate classification methods tailored to data with high dimensionality, numerous categories (and few repetitions). Cinematographic motion pictures are convenient *naturalistic* stimuli for studying complex (quasi-natural) perceptual processes by fMRI. They evoke a diverse yet robust and reproducible fMRI response throughout large visual areas in the lower occipital and temporal cortices. In a comparison of common classifiers average classification accuracies above 90% were achieved by the most successful combination of trial averaging, PCA and LDA. This is remarkably high, considering the relatively large number of classes (150) used in this study. However, absolute classification rates are not too meaningful, since they depend on many factors such as feature selection, variance normalization and other pre-processing of the data. Nevertheless, high classification rates may translate into high sensitivity for the detection of various cognitive and perceptual processes. The optimization of classification algorithms also reveals properties of the fMRI signal: Best classification results are supported by a global fROI, indicating that information about visual stimulus identity is not local but distributed throughout occipital and temporal cortices. Interestingly, the spatial and temporal patterns of the dominant PCA components are similar between subjects and reminiscent of functional connectivity networks.

## Author contributions

HM, Design of study and experimental procedures, data acquisition and analysis, interpretation of results, preparation of manuscript and figures. JDZ, Experimental design and data acquisition, interpretation of results, preparation of manuscript. JD, Study design, interpretation of results, and manuscript preparation.

### Conflict of interest statement

The authors declare that the research was conducted in the absence of any commercial or financial relationships that could be construed as a potential conflict of interest.

## Footnotes

^1^Details available upon request via https://amri.ninds.nih.gov/hMvCla.html

## Abbreviations

BOLD: blood oxygenation level dependent (fMRI)
BW: (readout) bandwidth (MRI parameter)
FA: flip angle (MRI parameter)
fMRI: functional MRI
FWHM: full width at half maximum
GLM: general linear model
GNB: Gaussian Naïve Bayes classifier
GRAPPA: (MRI parallel imaging technique)
HRF: hemodynamic response function
LDA: Linear Discriminant Analysis classifier
ML: machine learning (algorithm)
MRI: magnetic resonance imaging
MVPA: multivariate pattern analysis
NNE/C: nearest-neighbor classifier with Euclidean/Correlation distance metric
NME/C: nearest-mean classifier with Euclidean/Correlation distance metric
PC: principal component
PCA: principal component analysis
(f)ROI: (functional) region of interest
SNR: signal-to-noise ratio
SD: standard deviation
SVD: singular value decomposition
SVM: support vector machine
TE: echo time (MRI parameter)
TR: (volume) repetition time (MRI).
