# Supplementary material for: Linear Discriminant Analysis Achieves High Classification Accuracy for the BOLD fMRI Response to Naturalistic Movie Stimuli
Source: Front Hum Neurosci. 2016 Mar 31;10:128. doi: 10.3389/fnhum.2016.00128 (PMC4815557; doi:10.3389/fnhum.2016.00128)
Supplement: Supplementary file 1 [file DataSheet1.docx]

*Supplementary Material*

*Linear Discriminant Analysis* achieves high classification accuracy for the BOLD fMRI response to naturalistic movie stimuli.

H. Mandelkow^1,a^ , J. de Zwart^1^, J. Duyn^1^

1. Advanced MRI Section, Laboratory of Functional and Molecular Imaging, National Institute of Neurological Disorders and Stroke, National Institutes of Health, Bethesda, MD, USA
2. *Correspondence to:* [*Hendrik.Mandelkow@nih.gov*](mailto:Hendrik.Mandelkow@nih.gov)*, 10 Center Dr. 10-B1D728, Bethesda, MD 20892-1065, USA*

| Figure 6 LDA with 64 principal components achieved maximal classification accuracy.  Mean classification rates (y-axis) depended on the number of input features (x-axis), the number of principal components (8,16,…,128) and the similarity metric: cross-correlation (NMC), Euclidean (NME), normalised Euclidean (GNB) and the Mahalanobis distance (LDA), see Table 1. |
| --- |
|  |

| Figure 7 Within-session classification rates (aa) are higher than between-session (ab)  Odd/even bars (ab/aa) represent intER- and intRA-session classification rates for the two nearest-neighbour classifiers in Figure 2 (NNE, NNC) averaged over sessions and subjects. Since within-session classification rates (NNEaa, NNCaa) are systematically higher than between-session (NNEab, NNCab), only the latter were used in the main comparison (Figure 2). |
| --- |
|  |
